# Evaluation of Chronic Rhinosinusitis Symptoms' Severity Following COVID-19 Infection: A Retrospective Analysis

**DOI:** 10.7759/cureus.38517

**Published:** 2023-05-03

**Authors:** Ahmad Alroqi, Mashal B Abaalkhail, Nawaf Albuhayjan, Jehad Alorainy, Mohammed Jomah, Saud Alromaih, Khalifa Binkhamis

**Affiliations:** 1 Otolaryngology-Head & Neck Surgery, King Saud University, Riyadh, SAU; 2 Pathology, King Saud University, Riyadh, SAU

**Keywords:** health-related quality of life (hrqol), snot-22, intranasal corticosteroids, crs, covid-19

## Abstract

Objectives

This study aims to compare the severity of chronic rhinosinusitis (CRS) symptoms pre- and post-COVID-19 infection and estimate the impact of the COVID-19 pandemic on the use of intranasal corticosteroids (ICS) among adult CRS patients.

Methods

This was an observational retrospective cohort study conducted at King Abdulaziz University Hospital, Riyadh, Saudi Arabia, between July 2022 and October 2022. Adult CRS patients with sino-nasal outcomes test-22 (SNOT-22) scores documented prior to March 2020, marking the occurrence of Saudi Arabia's initial reported case of COVID-19, were requested to complete the SNOT-22 questionnaire following COVID-19 infection. A comparison was subsequently made between the two scores obtained.

Results

The study enrolled a total of 33 patients, with 16 assigned to the control group and 17 with a history of COVID-19 infection. The mean age of the patients was 43 years, and the majority (52%) were males. Statistical analysis did not reveal any statistically significant differences in the total SNOT-22 scores or domain-level scores between the two groups. Furthermore, the use of ICS during the COVID-19 pandemic did not show any significant associations, except for patients with asthma, where 80% of them used ICS during the pandemic (p=0.0073).

Conclusion

There was no statistically significant disparity observed in the SNOT-22 scores between patients who tested positive for COVID-19 and those who did not. The use of corticosteroids during the COVID-19 pandemic was found to be more prevalent in this study compared to previous studies conducted before the pandemic, particularly among patients with asthma. The use of ICS during the pandemic was not associated with the presence of polyps, functional endoscopic sinus surgery (FESS), allergic rhinitis, or eczema.

## Introduction

Chronic rhinosinusitis (CRS) is a multifaceted inflammatory condition with diverse phenotypes and endotypes [1]. Despite several studies on the association between COVID-19 and CRS, evidence remains limited on this topic [2]. Studies conducted in Iran and the USA revealed no increase in COVID-19 infection rates among CRS patients [3,4], while a case-control study in Spain suggested that CRS may be linked to a higher risk of prolonged viral shedding [5]. However, the presence of SARS-CoV-2 RNA does not necessarily correlate with infectivity, underscoring the need for further prospective trials to better define risk factors for prolonged viral shedding. Moreover, CRS has not been associated with complications such as acute respiratory distress syndrome, mechanical ventilation, or extended hospitalization in COVID-19 patients [5,6]. In terms of the impact of the COVID-19 pandemic on CRS patients, a recent study reported significantly lower average sino-nasal outcomes test-22 (SNOT-22) scores in CRS patients after one year from the onset of the pandemic compared to pre-pandemic scores [3]. However, this study did not consider the baseline CRS symptoms before COVID-19 infection and their comparison to the post-COVID-19 status. As highlighted in the position paper on the management of CRS during the COVID-19 pandemic by the European Academy of Allergy & Clinical Immunology (EAACI), intranasal corticosteroid (ICS) administration is considered safe and should be continued [7]. A retrospective study investigated the role of preoperative intranasal steroid irrigation as a non-surgical alternative during the COVID-19 era and found a significant improvement in Lund-Mackay and SNOT-22 scores, with 64.4% of CRS patients no longer requiring endoscopic sinus surgery [8]. However, a cohort study reported that ICS usage was associated with a higher risk of SARS-CoV-2 infection and severe outcomes in CRS patients compared to those not treated with ICS [9]. There is currently a paucity of literature investigating the changes in CRS symptoms before and after COVID-19 infection. Given the ongoing debates surrounding the use of steroids in the context of the COVID-19 pandemic, particularly as ICS are commonly employed in CRS medical management, this study seeks to assess the effects of COVID-19 infection on CRS symptoms and the potential influence of the pandemic on ICS utilization among patients with CRS.

## Materials and methods

Study design

This study employed an observational retrospective cohort design and was conducted at King Abdulaziz University Hospital in Riyadh, Saudi Arabia, from July 2022 to October 2022. The data were obtained from adult patients diagnosed with CRS who had documented SNOT-22 scores recorded before March 2020, which marked the onset of the COVID-19 pandemic in Saudi Arabia.

Subjects

The diagnosis of CRS in this study followed the criteria set forth by the European Position Paper on Rhinosinusitis and Nasal Polyps (EPOS), which defines CRS as the presence of two or more symptoms lasting for at least 12 weeks, with at least one symptom being nasal blockage/obstruction/congestion or nasal discharge (anterior/posterior nasal drip), ± facial pain/pressure ± reduction or loss of smell [10]. In addition, CT findings indicative of CRS were also required for inclusion, with CT findings quantified and interpreted using Lund-Mackay scoring (LMS), which assigns a maximum score of 24 or 12 per side [11]. An LMS score of 5 or higher was considered indicative of CRS. Exclusion criteria included pediatric patients (<14 years old), patients with cystic fibrosis, gross immunodeficiency, congenital mucociliary problems, non-invasive fungal balls and invasive fungal disease, systemic vasculitis, and granulomatous diseases, as well as patients with a history of cocaine abuse or cancer. The CRS patients with SNOT-22 scores less than 8 before March 2020 were also excluded to account for potential incorrect filing or documentation. To control for time-related confounding factors, subjects were categorized into cases defined as those with a confirmed history of COVID-19 infection by reverse transcription polymerase chain reaction (RT-PCR), and controls that included those who had no history of COVID-19 infection.

Data collection

Adult CRS patients with SNOT-22 scores documented prior to March 2020 were requested to complete the SNOT-22 questionnaire again following COVID-19 infection. All subjects were identified through electronic health records and subsequently contacted via phone calls and requested to complete the electronic questionnaire. Responses were directly recorded on an electronic data sheet to ensure accuracy and reliability. The study ensured the privacy and confidentiality of all participants, and informed consent was obtained from each participant. The validated Arabic version of SNOT-22, a 22-item chronic rhinosinusitis-specific quality-of-life (QoL) instrument, was utilized [12]. The SNOT-22 survey items were scored using a Likert scale, with scores ranging from 0 ("No problem") to 5 ("Problem as bad as it can be"), reflecting the increasing severity of symptoms. Higher scores on the SNOT-22 survey items indicate worse patient functioning or symptom severity, with a total score range of 0-110. In addition to the standard SNOT-22 questions, this study included additional questions about the patient's medical history and steroid use during the COVID-19 pandemic. The protocol of this study was approved by the College of Medicine Institutional Review Board, King Saud University, Riyadh, Saudi Arabia (approval no. E-22-6987).

Statistical analysis

The numerical data are described as mean and standard deviations (a normality check was conducted with the Shapiro-Wilk test and the p-value was >0.05), while categorical data are described as frequency and percentages. A p-value of <0.05 and 95% confidence interval (CI) were used to report the statistical significance and precision of the results. The t-test for unpaired groups was used to test the difference in the means of the SNOT scores between the cases and the controls. The chi-square test was used to test the associations between the use of ICS during the COVID-19 pandemic and demographic characteristics. The statistical analysis was performed using Stata-17 (StataCorp LLC, College Station, TX, USA).

## Results

In this study, a total of 101 patients with CRS were initially enrolled. However, after applying the pre-determined eligibility criteria, only 33 subjects were deemed suitable for inclusion in the study. This included 17 cases and 16 controls. The average age of the patients was 43 years, and the majority (52%) were male. The two groups were similar in terms of their baseline characteristics, except for the presence of polyps, which was found to be significantly higher in the control group (p=0.009), and functional endoscopic sinus surgery (FESS) (p=0.029). A summary of the baseline characteristics can be found in Table 1. Table 2 summarizes the comparison of the SNOT-22 scores between the cases and the controls. There was no statistically significant difference between the two groups in the total score or at the individual domain level. During the COVID-19 pandemic, it was observed that nearly half (45%) of the patients with CRS did not use ICS. Furthermore, an analysis was conducted to investigate the association between the use of ICS during the pandemic and the presence of polyps, FESS, allergic rhinitis, eczema, and asthma. The results indicated that there was no significant association between ICS use and these factors, with the exception of asthma. Among patients with asthma, 80% used ICS during the COVID-19 pandemic (p=0.0073). These findings are presented in Table 3.

**Table 1:** Demographics SD: Standard deviation, LMS: Lund-Mackay scoring, FESS: Functional endoscopic sinus surgery

Variables	Total (n=33)	Control (n=16)	Case (n=17)	p-value
Age (mean ± SD)	43 ± 16	48 ± 18	38 ± 14	0.09
Males, n (%)	17 (52)	8 (50)	9 (53)	0.87
Total LMS (mean ± SD)	13 ± 6	13 ± 5	14 ± 7	0.48
Polyps, n (%)	24 (73)	15 (94)	9 (53)	0.009
FESS				0.029
No	2 (6)	0 (0)	2 (12)	
Yes, before COVID-19 pandemic; n (%)	24 (73)	15 (94)	9 (53)	
Yes, after COVID-19 pandemic; n (%)	7 (21)	1 (6)	6 (35)	
Allergic rhinitis, n (%)	26 (79)	12 (75)	14 (82)	0.61
Eczema, n (%)	2 (6)	1 (6)	1 (6)	0.96
Asthma, n (%)	15 (45)	10 (63)	5 (29)	0.056

**Table 2:** Comparison of SNOT-22 scores between cases and controls SD: Standard deviation, SNOT: Sino-nasal outcomes test

Variables	Case	Control	p-value
Difference in nasal domain of SNOT score (mean ± SD)	−0.74 ± 13.89	0.06 ± 13.83	0.8651
Difference in facial domain of SNOT score (mean ± SD)	−0.41 ± 4.41	1.43 ± 2.55	0.1497
Difference in sleep domain of SNOT score (mean ± SD)	1.82 ± 7.47	−0.06 ± 6.90	0.4566
Difference in emotional domain of SNOT score (mean ± SD)	1.41 ± 9.86	−0.06 ± 8.01	0.6398
Difference in total SNOT score (mean ± SD)	2.05 ± 32.18	1.37 ± 27.47	0.948

**Table 3:** ICS use during the COVID-19 pandemic ICS: Intranasal corticosteroids, FESS: Functional endoscopic sinus surgery

Variables	Did not use ICS	Used ICS	p-value
Presence of polyps			0.4755
Absent, n (%)	5 (55.56)	4 (44.44)	
Present, n (%)	10 (41.67)	14 (58.33)	
FESS			0.2817
No, n (%)	1 (50)	1 (50)	
Yes, before COVID-19 pandemic; n (%)	9 (37.5)	15 (62.5)	
Yes, after COVID-19 pandemic; n (%)	5 (71.43)	2 (28.57)	
Allergic rhinitis			0.1200
Absent, n (%)	5 (71.43)	2 (28.57)	
Present, n (%)	10 (38.46)	16 (61.54)	
Eczema			0.8940
Absent, n (%)	14 (45.16)	17 (54.84)	
Present, n (%)	1 (50)	1 (50)	
Asthma			0.0073
Absent, n (%)	12 (66.67)	6 (33.33)	
Present, n (%)	3 (20)	12 (80)	
Comorbidities			0.0921
Absent, n (%)	4 (80)	1 (20)	
Present, n (%)	11 (39.29)	17 (60.71)	
Total, n (%)	15 (45)	18 (55)	0.11
Cases, n (%)	10 (59)	7 (41)	
Controls, n (%)	5 (32)	11 (68)	

## Discussion

The study assessed changes in symptom severity in participants with CRS before and after COVID-19 infection, as well as the impact of the pandemic on the use of ICS in adults with CRS. The severity of CRS symptoms was measured using the SNOT-22 questionnaire, which measures health-related QoL in patients with CRS. The results showed no statistically significant difference in SNOT-22 scores before and after COVID-19 infection when the four domains (nasal, facial, sleep, and emotional) were evaluated separately. Additionally, there was no significant difference in total SNOT-22 scores, and the mean difference in SNOT-22 scores before and after the COVID-19 pandemic was below the minimal clinically important difference (MCID) for the validated Arabic SNOT-22 questionnaire [12]. This indicates that COVID-19 infection did not have a significant effect on the severity of CRS symptoms. This finding is supported by another study, although with a slightly different sample, which also showed no statistical or clinically significant difference in SNOT-22 scores between COVID-19 positive and negative CRS patients. However, that study did report a statistically significant difference in SNOT-22 scores before and after the COVID-19 pandemic, but the change was not clinically significant as it was below the MCID cutoff [13]. Time confounder is the most likely reason for the statistically significant results they found when comparing the pre- and post-COVID-19 pandemic SNOT-22 score. Therefore, a control group was included in the present study to eliminate the time confounder and accurately assess whether COVID-19 infection had any effect on CRS symptom outcomes.

The use of ICS, which is the therapeutic standard for CRS, was also evaluated in this study. The EAACI's position paper on the management of CRS during COVID-19 suggests that corticosteroid administration is safe and should be continued as it may decrease virus-induced inflammation in the upper airways, and reduce the duration and severity of respiratory symptoms [7,14]. However, a Korean study reported that corticosteroid use was associated with a greater risk of SARS-CoV-2 infection and severe outcomes in CRS patients compared to those not treated with corticosteroids [9]. This indicates a controversy regarding ICS use in the COVID-19 era [9,14-16]. The present study found that only 55% of participants used ICS during the COVID-19 pandemic. This prevalence of ICS use was higher than in previous studies conducted in the UK and Canada before the COVID-19 pandemic, where only 15% and 20% of patients used corticosteroids, respectively [17,18]. Additionally, the study evaluated the potential association between corticosteroid use and various comorbid conditions. The majority (80%) of patients with asthma were found to have used corticosteroids during the COVID-19 pandemic, suggesting that patients with asthma were more likely to continue using corticosteroids during the pandemic. However, no significant association was found between corticosteroid use and the presence of polyps, FESS, allergic rhinitis, or eczema during this period.

This study had several limitations including the small sample size due to strict eligibility criteria, resulting in the exclusion of a majority of respondents. However, these criteria were implemented to ensure internal validity by minimizing possible confounders. Efforts were made to address the time confounder by including a control group for comparison. It should be noted that a higher number of participants in the control group had nasal polyps and underwent FESS before the pandemic compared to cases, which could potentially introduce biased findings. Nevertheless, despite these limitations, the findings of this study contribute to the existing literature on CRS and COVID-19. Further large-scale studies are warranted to comprehensively evaluate the possible effects of COVID-19 infection on CRS symptoms and outcomes.

## Conclusions

In conclusion, the results of this study suggest that COVID-19 infection did not have a statistically or clinically significant effect on the severity of CRS symptoms as measured by the SNOT-22 questionnaire. The use of corticosteroids during the COVID-19 pandemic was found to be more prevalent in this study compared to previous studies conducted before the pandemic, particularly among patients with asthma. The use of ICS during the pandemic was not associated with the presence of polyps, FESS, allergic rhinitis, or eczema. Overall, the findings of this study provide valuable insights for clinicians managing CRS patients post-COVID-19 infection. Further large-scale studies are needed to better understand the potential effects of COVID-19 on CRS symptoms and outcomes.
